# Healthcare providers’ hospital breastfeeding practices during the COVID-19 endemic and associated factors in Thailand: a cross-sectional study

**DOI:** 10.1186/s12912-024-02498-4

**Published:** 2024-11-17

**Authors:** Nongyao Lawin, Sasitara Nuampa, Chananchida Somsuk, Sutthisak Srisawad, Kasem Raungrongmorakot, Sukwadee Ketsuwan

**Affiliations:** 1 Obstetric & Gynecology Nursing Department, HRH Princess Maha Chakri Sirindhorn Medical Center, Srinakharinwirot University, Nakhon Nayok, Thailand; 2 Department of Obstetric and Gynaecological Nursing, Faculty of Nursing, Mahidol University, Bangkok, 10700 Thailand; 3 Bureau of Health Promotion, Department of Health, Nonthaburi, Thailand; 4 Division of Research Promotion and Development, Faculty of Nursing, Mahidol University, Bangkok, Thailand; 5 Department of Obstetrics & Gynecology, Faculty of Medicine, Srinakharinwirot University, Nakhon Nayok, Thailand

**Keywords:** Healthcare professionals, Breastfeeding practice, COVID-19, Endemic, Thailand

## Abstract

**Background:**

During COVID-19, healthcare providers were limited in their ability to provide breastfeeding support while women encountered breastfeeding difficulties. Enhancing appropriate breastfeeding care practices among healthcare providers in hospitals may improve the safety of breastfeeding during an endemic. However, little is known about the breastfeeding care practices by healthcare providers and associated factors during the endemic impact.

**Objective:**

To investigate the effect of the endemic on breastfeeding care practices by healthcare providers in hospitals and examine their associated factors in Thailand.

**Methods:**

A descriptive comparative design was conducted through an online survey with 350 healthcare providers across five regions of Thailand between January and March 2022. The convenience sampling was used to recruit healthcare providers who had at least two years of experience supporting breastfeeding practices and were full-time working in the obstetric and pediatric departments of public tertiary hospitals. Analysis of variance and the independent t-test with relevant statistical corrections were utilized for comparisons of associated factors on breastfeeding care practices in healthcare providers.

**Results:**

The mean breastfeeding care practices in hospitals during the COVID-19 endemic by healthcare providers was 39.17 (SD = 4.64, range 23 to 50). Four factors were statistically significant differences in breastfeeding care practices score, including work position (F = 7.03, df = 2.0, *p* = 0.001), types of COVID-19 vaccination (F = 6.95, df = 2, *p* = 0.001), education (F = 4.78, df = 2, *p* = 0.009), and monthly family income (F = 4.25, df = 3, *p* = 0.006), respectively. In addition, dose of COVID-19 vaccination and types of COVID-19 vaccination were significantly associated with individual breastfeeding support in hospitals (*p* < 0.05).

**Conclusions:**

Healthcare providers’ breastfeeding care practices in hospitals during the COVID-19 endemic were mostly at a moderate level in the Thai context. Hospital policy for maternal and child health support should strongly recommend the effective and safe practice of breastfeeding to encourage mothers to continue their breastfeeding duration.

**Supplementary Information:**

The online version contains supplementary material available at 10.1186/s12912-024-02498-4.

## Introduction

The COVID-19 pandemic has been a major health threat over the past 2–3 years. In late 2021, the Omicron variant emerged with mutations that greatly increased its transmissibility compared to earlier strains [1, 2]. In Thailand, the first confirmed case of the Omicron variant was reported in December 2021. By January 2022, the number of infections had surged to almost 2.4 million cases, with approximately 22,291 cumulative deaths [3]. This figure dramatically increased to 4.2 million cases and 34,425 accumulated deaths by April 2022 [4]. This alarming situation raised concerns about the capacity of the Thai healthcare system, which faced the risk of collapsing during the Omicron pandemic. Despite these challenges, the Ministry of Public Health declared in January 2022 that the COVID-19 outbreak had transitioned into an endemic disease [5]. Concurrently, efforts were underway to mitigate the impact through vaccination strategies. As of January 2022, vaccination statistics for Thailand indicated that approximately 52 million people (78%) and 48 million people (72%) had received the first and second doses of the COVID-19 vaccine, respectively [6, 7]. However, the third dose had been administered to only about 14 million people (21%), indicating a substantial portion of the at-risk population remained without this additional level of protection [8].

Research conducted thus far suggests that antibodies of the virus that causes the COVID-19 found in breast milk exhibit a robust immunological response against the virus [9, 10]. Both COVID-19 vaccination and natural infection with the virus induce the production of antibodies in the mother. These antibodies neutralize the virus by binding to various regions of the spike glycoprotein and are also present in breast milk [11, 12]. Global recommendations advocate for women to continue breastfeeding to enhance the health and immunity of their infants during the pandemic [13, 14]. Despite the encouragement to continue breastfeeding, certain hospital practices, especially those concerning mother-newborn contact, raise concerns about the potential transmission of the COVID-19 from mother to newborn [15]. In a study by Merewood et al. [16], which assessed data from 124 hospitals across 22 nations, it was revealed that 72% of hospitals based their policies on their country’s national guidelines, while 31% followed WHO guidelines. The study found that 38% of hospitals prohibited all visitors for birthing women, 19% shortened postpartum stays, and 6% recommended formula feeding instead of breastfeeding for women under investigation. At the onset of the pandemic, rapidly evolving guidelines and conflicting recommendations regarding the care of infants born to mothers suspected of or confirmed to have COVID-19 infection led to unnecessary separation of mothers and infants [17]. A survey of Mississippi birthing hospitals revealed varied sources for maternity care policies and differences between institutions. During the pandemic, breastfeeding rates plateaued, while skin-to-skin contact and rooming-in decreased [18]. During the COVID-19 pandemic, postpartum mothers tended to receive more breastfeeding support from informal social networks than from healthcare providers, which impacted the success of exclusive breastfeeding [19]. Less than half of healthcare providers were aware of recommendations regarding breastfeeding practices during the COVID-19 pandemic [20]. Inadequate breastfeeding support was associated with the discontinuation of exclusive breastfeeding during the pandemic [19].

Currently, we are embedded in the COVID-19 pandemic crisis until an endemic situation. The previous studies disclosed vital changes and some challenges for breastfeeding support services through healthcare providers’ practices. However, breastfeeding care practices in hospitals by healthcare providers in the Thai context during the COVID-19 endemic were limited. This study investigated the effect of the COVID-19 endemic on healthcare providers’ breastfeeding care practices in Thailand.

The specific aims were to answer the following research questions: (1) What is the situation of hospital breastfeeding practices in healthcare providers during the COVID-19 endemic in Thailand? (2) What factors are associated with differences in hospital breastfeeding care practices levels by healthcare providers during the COVID-19 endemic in Thailand? and (3) Are the COVID-19 vaccination dose and types of healthcare providers associated with hospital breastfeeding care practices during the COVID-19 endemic in Thailand?

## Materials and methods

### Study design and participants

A descriptive comparative design was conducted through an online questionnaire survey in five regions in Thailand, namely the northern, southern, central, eastern, and north-eastern regions. Five provinces were selected by simple random sampling from each region for the recruitment of study participants and data collection. The advance-level government hospitals (tertiary hospitals) of each province were recruited to survey. The convenience sampling was used to recruit healthcare providers who were experienced to support breastfeeding for at least two years and full-time working in the obstetric and pediatric department in public tertiary hospitals, and able to access an online survey to participate in this study. Healthcare providers in this study consisted of obstetricians, pediatricians, obstetrical nurses, pediatric nurses, and nurse assistants. Data were collected from January to March 2022. This study calculated the sample size using the G*Power program (version 3.1.9.4) by comparing the means of analysis of variance (ANOVA), with medium effect size, power of test 0.95, and alpha 0.05; the required sample size will be at least 332. The sample size was set to be 350, which was approximately 5% more than the calculation to prevent missing data.

### Data collection

The poster and infographic were distributed as invitations to participate in the study without any coercion. Participants who were willing to join the study were able to scan the QR code for the consent form and questionnaire. All participants completed an online informed consent form prior to the beginning of data collection. The informed consent explained the study purpose, assured confidentiality of their identity, and gave them the right to refuse to participate. In an online survey, 350 participants completed the questionnaire. Despite an initial response rate of 92.3%, the research team continued data collection until reaching the target of 350 completed cases.

### Ethical approval

This study was conducted in accordance with the Declaration of Helsinki and approved by the Human Research Ethic Committee of Srinakharinwirot University, Thailand (SWUEC/E-044/2564). The written electronic informed consents were administrative prior data collection.

### Measures

This study consisted of two parts of a self-administered online survey, including personal information questionnaires with 11 items and Healthcare Providers’ Hospital Breastfeeding Practices During COVID-19 (HCPBF-COVID19) questionnaires for healthcare providers with 10 items. The personal information questionnaires and the HCPBF-COVID19 were developed by researchers according to the literature review about healthcare professional breastfeeding practices, COVID-19 breastfeeding guideline [15, 16, 19, 20]. Regarding the HCPBF-COVID19, the content was divided into two parts: policy management for breastfeeding care practices (items 1, 3, 5, 6, and 7) and safety practices in breastfeeding care (items 2, 4, 8, 9, and 10). The questionnaire responses were 5-point Likert scales ranging from 1 (strongly disagree) to 5 (strongly agree), range 10–50, with higher scores indicating a high level of breastfeeding care practices in hospitals to provide during the COVID-19 endemic (Supplementary File 1). A high level of breastfeeding care practices in the hospital indicates that healthcare providers implement safety practices to prevent COVID-19 transmission during breastfeeding support, and that hospital policies effectively facilitate appropriate breastfeeding support in alignment with global recommendations [21–23].

The measurements of this study assessed validity and reliability. A Content Validity Index (CVI) was calculated, including both item-level (I-CVI) and scale-level (S-CVI) indices, to evaluate each item’s relevance. Content validity refers to the extent to which the instrument comprehensively measures the construct of interest, which in this case pertains to hospital breastfeeding practices and the COVID-19 breastfeeding guidelines. Three experts in breastfeeding and COVID-19 infection management—two nursing and midwifery instructors and an administrative nurse from the postpartum unit—validated the instruments to ensure conceptual accuracy and clarity, resulting in an I-CVI and S-CVI of 1. After modifying the content based on their recommendations, the revised version of the HCPBF-COVID19 was assessed for internal consistency reliability using the coefficient alpha index. A pilot test involving 30 healthcare providers was conducted, and data were analyzed using SPSS software, yielding a Cronbach’s alpha of 0.84, indicating acceptable reliability [24]. Finally, the reliability was confirmed after data collection from 350 healthcare providers, resulting in a Cronbach’s alpha of 0.79.

### Data analysis

The Statistical Package for Social Sciences (SPSS) version 20 was used to analyze the data. Descriptive statistics were presented as mean and standard deviation (SD). Breastfeeding care practices of healthcare providers between different groups were compared with ANOVA tests and t-tests. In addition, the doses and types of COVID-19 vaccination were important contributors influencing endemic work policies. These were analyzed using HCPBF-COVID items across different groups of these factors through an ANOVA test. Significance was set at *p* < 0.05 (significance level 95%).

## Results

### The characteristics of healthcare providers

The majority of healthcare professionals (99.1%) were female, with an average age of 38.96 years (SD = 10.26; min-max = 18–59). 56.3% of them had a single status. Of them, 92% identified as Buddhists. In terms of education, the majority of them (81.7%) held a diploma or bachelor’s degree, while three healthcare providers graduated in doctoral degree (0.86%). They made the average family income of 45,358 baht (SD = 61,058) each month. This study collected work information from three groups of hospital staff members who support and promote breastfeeding: physicians (2.9%), nurses (77.4%), and nursing assistants (19.7%). Their employment experience ranged from 2 to 39 years, with an average of 13.01 years (SD = 10.34). An average of 2.88 doses (SD = 0.34; min-max = 1–3) of the COVID-19 vaccine were administered to all 350 providers, the majority of whom received mixed types, as was presented in Table 1.

### The factors are associated with differences in hospital breastfeeding care practices levels during the COVID-19 endemic

The hospital breastfeeding practice scores were separated into three levels of 25 and 75 percentile, including low level (< 36 scores), moderate level (36–42 scores), and high level (> 42 scores). More than half of them had a moderate level (53.7%), while one of four had a low level (26.6%), and 19.7% had a high level. The average breastfeeding practice in hospitals during COVID-19 among healthcare providers was 39.17 (SD = 4.64) with a range of scores between 23 and 50.

Table 1 shows the personal information, working experiences, the COVID-19 vaccination, and those factors associated with breastfeeding practice in hospitals during the COVID-19 endemic among healthcare providers in five regions of Thailand. Four factors were statistically significant differences in breastfeeding care practice score: work position (*F* = 7.03, *df* = 2.0, *p* = 0.001), types of COVID-19 vaccination (*F* = 6.95, *df* = 2, *p* = 0.001), education (*F* = 4.78, *df* = 2, *p* = 0.009), and monthly family income (*F* = 4.25, *df* = 3, *p* = 0.006).

**Table 1:** Healthcare provider’s characteristic data, working information, COVID-19 vaccination, and association with hospital breastfeeding practice during COVID-19 (*N* = 350)

	*n* (%)	Hospital Breastfeeding Care Practice		
		Min - Max	Mean ± SD	*p* value
**Sex** ^a^ Females Males	347 (99.1) 3 (0.9)	23–50 33–50	39.14 ± 4.61 42.33 ± 8.62	0.236
**Age (year)**				
< 26	37 (10.6)	33–50	40.13 ± 4.96	0.069
26–40	160 (45.7)	28–50	39.59 ± 4.56	
41–55	124 (35.4)	30–50	38.71 ± 4.20	
≥ 56	29 (8.3)	23–50	37.66 ± 5.96	
**Marital status**				
Single	197 (56.3)	23–50	39.29 ± 4.46	0.710
Married	130 (37.1)	28–50	39.09 ± 4.95	
Divorce	23 (6.6)	30–50	38.47 ± 4.37	
**Religion**				
Buddhist	322 (92.0)	28–50	39.23 ± 4.56	0.624
Muslim	23 (6.6)	23–49	39.00 ± 6.51	
Christian	5 (1.4)	35–50	38.26 ± 5.41	
**Education**				
High school	38 (10.9)	35–50	40.97 ± 3.45	0.009*
Diploma/ Bachelor	286 (81.7)	28–50	39.08 ± 4.73	
Master’s degree or above	26 (7.4)	23–50	37.46 ± 4.50	
**Family income (month/baht)**				
< 15,000	55 (15.7)	30–50	40.92 ± 5.12	0.006*
15,001–30,000	116 (33.1)	28–50	39.09 ± 4.52	
30,001–50,000	113 (32.3)	28–47	38.25 ± 3.77	
>50,000	66 (18.9)	23–50	39.37 ± 5.39	
**Region**				
North	70 (20.0)	28–50	39.00 **±** 4.77	0.318
Central	70 (20.0)	28–50	38.67 ± 4.61	
East	70 (20.0)	28–49	38.91 ± 4.52	
North East	70 (20.0)	31–50	40.21 ± 4.79	
South	70 (20.0)	23–50	39.03 **±** 4.49	
**Work position**				
Pediatrician and Obstetrician	10 (2.9)	30–45	36.90 ± 5.97	0.001*
Nurse	271 (77.4)	23–50	38.80 ± 4.60	
Nurse assistant	69 (19.7)	30–50	40.89 ± 4.18	
**Work unit**				
Lactation clinic	17 (4.9)	23–44	38.82 ± 5.16	0.217
Well-baby clinic	7 (2.0)	32–44	38.42 ± 4.72	
Antenatal care unit	198 (56.6)	28–50	39.25 ± 4.71	
Delivery unit	34 (9.7)	29–49	37.52 ± 4.33	
Postpartum care unit	94 (26.9)	28–50	39.69 ± 4.46	
**Work experience (year)**				
2–4	83 (23.7)	30–50	39.75 ± 5.24	0.093
5–10	105 (30.0)	28–50	39.04 ± 4.44	
11–20	65 (18.6)	28–49	39.89 ± 4.18	
≥ 21	97 (27.7)	23–50	38.29 ± 4.51	
**Doses of COVID-19 vaccination**				
1 dose	2 (0.6)	42–43	42.50 ± 0.71	0.561
2 doses	37 (10.6)	30–50	38.89 ± 5.14	
3 doses	311 (88.8)	23–50	39.17 ± 4.59	
**Types of COVID-19 Vaccination**				
mRNA	13 (3.7)	30–41	35.84 ± 3.64	0.001*
Inactivated	109 (31.1)	23–50	38.35 ± 4.63	
Mixed	228 (65.1)	28–50	39.74 ± 4.57	

^*^P Value < 0.05, one way ANOVA Test, ^a^the independent t-test

### The hospital breastfeeding care practices and those associated with the doses and types of COVID-19 vaccination

**Fig. 1:**
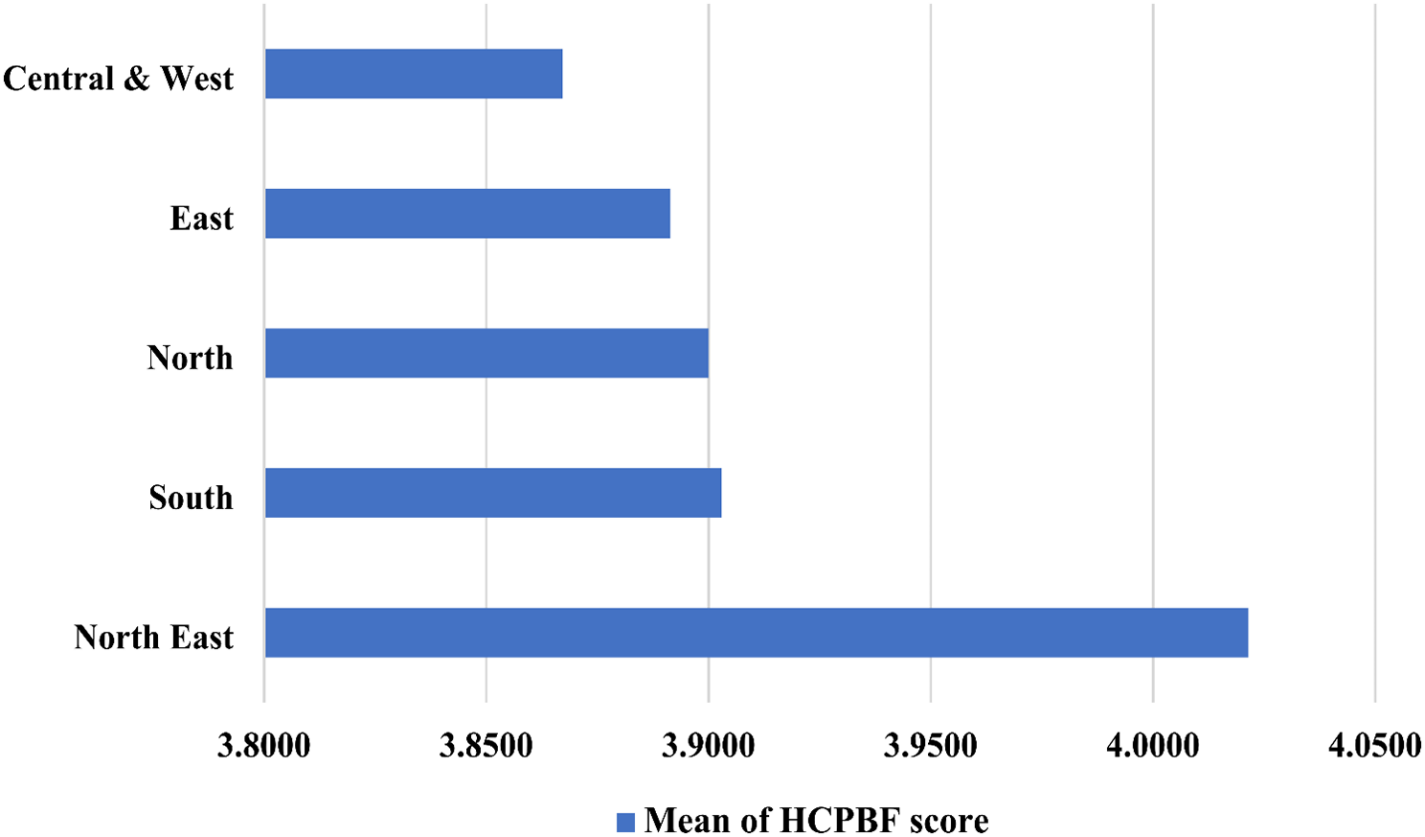
The means of healthcare providers’ breastfeeding care practices in hospitals divided by region

The average score of HCPBF-COVID had three items that were higher than 4, including the breastfeeding care practices about wearing a face mask (4.95 ± 0.22), washing hands (4.92 ± 0.34), and individual breastfeeding support (4.02 ± 0.79), respectively. While the enough staff to work on breastfeeding support was the lowest score (3.08 ± 0.94). Moreover, item analysis of HCPBF-COVID and those associated with COVID-19 vaccination presented five items were statistically significant differences in types of COVID-19 vaccinations: item 7 (*F* = 10.49, *df* = 2.0, *p* = 0.000), item 2 (*F* = 7.51, *df* = 2.0, *p* = 0.001), item 6 (*F* = 6.75, *df* = 2.0, *p* = 0.001), item 5 (*F* = 5.82, *df* = 2.0, *p* = 0.003), and item 3 (*F* = 3.65, *df* = 2.0, *p* = 0.027). While only one item was statistically significant differences in doses of COVID-19 vaccinations, that was item 2 (*F* = 8.51, *df* = 1.0, *p* = 0.037). The additional data was presented in Table 2. The average of hospital breastfeeding care practice during the COVID-19 endemic was divided by five regions consisting of the north-east region (4.02 ± 0.05), south region (3.91 ± 0.05), north region (3.90 ± 0.06), east region (3.89 ± 0.05), and central and west (3.86 ± 0.06) (Fig. 1).

**Table 2:** The descriptive data of hospital breastfeeding practices during COVID-19 through item analysis and associated with COVID-19 vaccination

No	Healthcare Providers’ Hospital Breastfeeding Care Practices	Mean ± SD	Dose of Covid-19 Vaccination (*p* value)	Types of Covid-19 Vaccination (*p* value)
1	I receive knowledge and training skills for preventing the COVID-19 while providing breastfeeding support	3.79 ± 0.83	0.070	0.524
2	I support mothers individually about breastfeeding	4.02 ± 0.79	0.037*	0.001*
3	My workplace is conducive to breastfeeding practices during the COVID-19	3.69 ± 0.94	0.730	0.027*
4	While working on breastfeeding during the COVID-19, I was given personal protective equipment in accordance with guidelines	3.95 ± 0.75	0.277	0.916
5	I have spent more time helping mothers with breastfeeding in each case compared to before the COVID-19 situation	3.39 ± 0.93	0.541	0.003*
6	The number of breastfeeding services in my organization increased during the COVID-19 situation	3.44 ± 0.90	0.488	0.001*
7	There are enough HCPs to work on breastfeeding support during the COVID-19	3.08 ± 0.94	0.389	0.000*
8	I wash my hands before and after touching all mothers when supporting breastfeeding practice	4.92 ± 0.34	0.215	0.165
9	I always wear a face mask when I work on breastfeeding	4.95 ± 0.22	0.945	0.091
10	I always wear face shield and face mask while working on breastfeeding	3.92 ± 1.05	0.695	0.169

^*^P Value < 0.05, one way ANOVA Test, HCPs; Healthcare providers

## Discussion

This study aimed to explore the impact of the COVID-19 endemic on breastfeeding care practices among healthcare providers in hospitals, and to identify factors associated with these practices. The findings reveal that most healthcare providers involved were obstetric and pediatric nurses, who supported breastfeeding primarily during antenatal and postpartum care. These results align with previous studies indicating that nurses were a significant formal support network for breastfeeding during the COVID-19 pandemic [19].

Furthermore, our study found that the majority of healthcare providers received three doses of the COVID-19 vaccine. However, one in five had received only 1–2 doses, despite three years passing since the onset of the pandemic. In Thailand, by the end of March 2022, approximately 1.3 million healthcare providers and 0.6 million healthcare workers had received their third dose of the COVID-19 vaccine [25]. The vaccines administered were primarily viral vector vaccines (AstraZeneca), mRNA vaccines (Pfizer), and inactivated vaccines (Sinovac), with many healthcare providers receiving mixed vaccine types [26], consistent with the findings of this study. The COVID-19 vaccination acceptance among healthcare workers is likely to influence their role in providing care. Nearly two-thirds of respondents in other studies indicated a willingness to receive the COVID-19 vaccine, primarily due to concerns about contracting the virus and working directly with COVID-19 patients [27]. However, vaccine hesitancy remained a challenge; for instance, only 14.3% of hesitant respondents in the United States were willing to accept a hypothetical booster dose [28]. Similar trends were observed in studies from India and Saudi Arabia, where reluctance to receive, the vaccine was attributed to doubts about its effectiveness (32%) and concerns about potential long-term side effects (31%) [29]. To address vaccine hesitancy and improve breastfeeding care practices, implementing personalized education, targeted risk communication, and tailored policy measures could help reduce the number of individuals unwilling to receive a booster shot.

This study found that breastfeeding care practices during the COVID-19 endemic were at moderate levels. However, this assessment primarily reflects two aspects: infection prevention behavior and hospital breastfeeding policies, both measured with strong reliability [24, 30]. The World Health Organization (WHO) and the United Nations Children’s Fund (UNICEF) have affirmed the Baby-Friendly Hospital Initiative (BFHI) as the most effective approach for ensuring maternal and newborn health during the pandemic. The benefits of breastfeeding, bonding, and closeness have been shown to outweigh the minimal risk of virus transmission [31]. Although hospital breastfeeding policies continue to evolve, the critical importance of breastfeeding remains unchanged. Exclusive breastfeeding in the first few days of life is particularly crucial, as colostrum provides essential bioactive and immune cells that protect the newborn [32]. Furthermore, breastfeeding during maternal COVID-19 infection has been shown to offer protective benefits for the newborn [33]. WHO also recommends Early Essential Newborn Care (EENC), including prolonged skin-to-skin contact, rooming-in, and exclusive breastfeeding, even for mothers with COVID-19, due to its strong association with improved maternal and newborn outcomes [31].

This study’s item analysis revealed that while healthcare providers were diligent in implementing safety measures to prevent infection transmission, they faced challenges in fully supporting breastfeeding policies. These difficulties were likely due to increased workloads. Previous research found that healthcare providers experienced significant stress and difficulty in supporting hospital breastfeeding practices during the pandemic, particularly when clear lactation policies were not in place [34]. Nurses often faced dilemmas related to both internal and external issues, including shifting hospital policies, vaccine management, and evolving guidelines [35]. The COVID-19 pandemic has placed an immense burden on the healthcare sector, increasing workloads and contributing to physical and mental strain among healthcare professionals [36]. To improve breastfeeding outcomes during endemic conditions, hospital policies should be strongly aligned with BFHI and EENC guidelines. Additionally, efforts should be made to balance healthcare staff workloads with breastfeeding care practices to ensure optimal maternal and newborn health.

This study also found statistically significant differences in breastfeeding care practices in hospitals based on factors such as work position, education level, family income, and type of vaccination. Nurses and nursing assistants—particularly nursing assistants—played an important role in providing breastfeeding support in hospitals. During the complex and evolving circumstances of COVID-19, physicians and nurses often assumed leadership roles, while novice nurses and nursing assistants were responsible for delivering fundamental healthcare. A previous survey conducted in India reported that nearly 80% of nursing assistants and nurses, and 58.33% of medical officers were aware that it is safe to provide expressed breast milk from COVID-19-suspected or positive mothers. Additionally, all nursing assistants, nurses, and medical officers were familiar with the hygiene recommendations for breastfeeding mothers who were suspected or confirmed to have COVID-19 [37].

While most nurses had a positive attitude toward breastfeeding during the COVID-19 pandemic, 64.5% demonstrated a low level of knowledge regarding the World Health Organization’s breastfeeding guidelines during the pandemic [38]. Novice nurses, in particular, were often not confident in supporting breastfeeding mothers due to their lack of experience and skills. These nurses expressed a need for frequent practice, access to role models, and additional support [39]. To improve breastfeeding care practices during the COVID-19 pandemic, it is essential to disseminate updated information through training programs and guidelines across all levels of healthcare. Providing breastfeeding training courses focused on effective and safe practices during the COVID-19 endemic is crucial for enhancing the competence and confidence of healthcare providers [20].

This study found that healthcare providers with lower levels of education and family income demonstrated significantly higher breastfeeding care practice scores. These demographic factors may be related to their work roles in providing breastfeeding support. Healthcare professionals play a crucial role in promoting and maintaining breastfeeding practices [40]. Numerous systematic reviews have shown that appropriate professional interventions increase breastfeeding initiation rates [41], and that professional support—particularly when provided by trained staff—can effectively prolong exclusive breastfeeding [42]. However, previous studies from low- and middle-income countries have reported insufficient overall breastfeeding knowledge among healthcare workers, particularly in areas such as breastfeeding during maternal illness and proper positioning [43]. The updated BFHI emphasizes the importance of enhancing healthcare providers’ competencies, counseling skills, and comprehensive breastfeeding knowledge to improve the quality of breastfeeding support.

Endemic viruses are constantly present and have a predictable spread that predictability allows health care systems and healthcare providers to prepare, adapt, and reduce loss of life [44]. Regarding breastfeeding care practices, one of the key components of maternity services is breastfeeding, which requires clear guidelines for consistent practices across settings. These guidelines were indeed prepared based on the best available evidence generated so far. So, generating representative and ongoing evidence remains a mainstay of a roadmap from this perspective [45, 46]. Therefore, hospital policy should be developed clearly to serve the standard of breastfeeding support during the COVID-19 endemic. Fostering a hospital culture that values breastfeeding and emphasizes the importance of human milk will be essential in motivating healthcare professionals. Healthcare providers must be familiar with the recommended guidelines and current evidence of global organizations to be able to provide practical, mother-sensitive, and person-centered support, particularly in the safety breastfeeding practice to encourage mothers to continue their breastfeeding duration.

The limitations of this study include the variation in participant characteristics due to the use of online survey strategies to recruit willing healthcare providers. Notably, the relatively small number of physicians in the sample may limit the generalizability of the findings to all healthcare providers. Additionally, the novel instruments employed in this study may not have captured the full scope of breastfeeding care practices during the endemic. Nonetheless, the study effectively assessed two key dimensions: adherence to hospital breastfeeding policies and the implementation of safety practices in breastfeeding care, both of which demonstrated strong reliability. However, future studies should incorporate a more comprehensive measurement of breastfeeding practices.

## Conclusions

Breastfeeding care practices in hospitals during the endemic in Thailand need significant improvement, particularly through the development of clear breastfeeding support policies that guide healthcare providers beyond safety regulations. Additionally, enhancing breastfeeding knowledge and skills for healthcare providers may be crucial for achieving positive breastfeeding outcomes. Clear guidelines on breastfeeding practices during an endemic should be disseminated to all healthcare providers, along with effective management of staff workloads to ensure adequate breastfeeding support. Moreover, the type and dosage of COVID-19 vaccinations influenced healthcare providers’ breastfeeding care practices in hospitals. In particular, personalized, timely, and adequate support was crucial for ensuring a successful start to breastfeeding for mothers and their infants. Further research should explore the relationship between breastfeeding knowledge, vaccination willingness among healthcare providers, and breastfeeding care and outcomes.

## Electronic supplementary material

Below is the link to the electronic supplementary material.

Supplementary Material 1

## Data Availability

The datasets generated and/or analysed during the current study are not publicly available due privacy protection and ethical obligations but are available (in deidentified form) from the corresponding author on reasonable request.
