# Supplementary material for: Healthcare providers’ hospital breastfeeding practices during the COVID-19 endemic and associated factors in Thailand: a cross-sectional study
Source: BMC Nurs. 2024 Nov 17;23:840. doi: 10.1186/s12912-024-02498-4 (PMC11569598; doi:10.1186/s12912-024-02498-4)
Supplement: Supplementary file 1 — Supplementary Material 1 [file 12912_2024_2498_MOESM1_ESM.pdf]

Survey Questionnaire

Healthcare Providers' Hospital Breastfeeding Practices during the COVID-19 Endemic in Thailand

Section 1 General Information Survey for Healthcare Providers

Instructions: Please place a checkmark (✓) in the parentheses next to the text corresponding to your preferred choice or fill in the blank space according to the truth.

1. Gender
   ( ) 1. Female
   ( ) 2. Male
2. Current age...........years......months
3. Occupation
   ( ) 1. Physician
      ( ) 1.1 Obstetrician
      ( ) 1.2 Pediatrician
   ( ) 2. Registered Nurse
   ( ) 3. Nurse Assistant
   ( ) 4. Other, specify..................
4. Marital Status
   ( ) 1. Single
   ( ) 2. Married
   ( ) 3. Widowed / Divorced / Separated
5. Religion
   ( ) 1. Buddhism
   ( ) 2. Christianity
   ( ) 3. Islam
   ( ) 4. Other, specify...............
6. Highest Education Level
   ( ) 1. Diploma
   ( ) 2. Bachelor's Degree
   ( ) 3. Master's Degree

( ) 4. Doctorate

( ) 5. Other, specify...

7. Family Income...................................... Baht/Month
8. Currently working in the department of...................... for................. years........... months
9. Experience in breastfeeding support services.................. years........... months
10. Have you received the COVID-19 vaccine?

( ) 1. No

( ) 2. Yes, vaccine name....................................Number of doses received................ doses

Date of dose 1..........................

Date of dose 2.........................

Date of dose 3..........................

Section 2 Healthcare Providers' Hospital Breastfeeding Practices During COVID-19 Questionnaire (BFHCP-COVID19)

Instructions: Please place a checkmark (✓) in the box that corresponds to your most preferred opinion.

No	Healthcare Providers' Hospital Breastfeeding Practices	Strongly disagree (1)	Disagree (2)	Neutral (3)	Agree (4)	Strongly agree (5)
1	I receive knowledge and training skills for preventing the COVID-19 while providing breastfeeding support					
2	I support mothers individually about breastfeeding					
3	My workplace is conducive to breastfeeding practices during the COVID-19					
4	While working on breastfeeding during the COVID-19, I was given personal protective equipment in accordance with guidelines					
5	I have spent more time helping mothers with breastfeeding in each case compared to before the COVID-19 situation					
6	The number of breastfeeding services in my organization increased during the COVID-19 situation					

7	There are enough HCPs to work on breastfeeding support during the COVID-19					
8	I wash my hands before and after touching all mothers when supporting breastfeeding practice					
9	I always wear a face mask when I work on breastfeeding					
10	I always wear face shield and face mask while working on breastfeeding					
